# Microfluidic preparation of polymer nanospheres

**DOI:** 10.1007/s11051-014-2626-5

**Published:** 2014-12-04

**Authors:** Israfil Kucuk, Mohan Edirisinghe

**Affiliations:** 1 Department of Mechanical Engineering, University College London, Torrington Place, London, WC1E 7JE UK; 2 Department of Metallurgical and Materials Engineering, Faculty of Engineering, Firat University, Elazig, 23279 Turkey

**Keywords:** Polymethylsilsesquioxane, Perfluorohexane, Microfluidics, Surface morphology, Nanopheres, Nanocarriers, Nanobiotechnology

## Abstract

In this work, solid polymer nanospheres with their surface tailored for drug adhesion were prepared using a V-shaped microfluidic junction. The biocompatible polymer solutions were infused using two channels of the microfluidic junction which was also simultaneously fed with a volatile liquid, perfluorohexane using the other channel. The mechanism by which the nanospheres are generated is explained using high speed camera imaging. The polymer concentration (5–50 wt%) and flow rates of the feeds (50–300 µl min^−1^) were important parameters in controlling the nanosphere diameter. The diameter of the polymer nanospheres was found to be in the range of 80–920 nm with a polydispersity index of 11–19 %. The interior structure and surfaces of the nanospheres prepared were studied using advanced microscopy and showed the presence of fine pores and cracks on surface which can be used as drug entrapment locations.

**Electronic supplementary material:**

The online version of this article (doi:10.1007/s11051-014-2626-5) contains supplementary material, which is available to authorized users.

## Introduction

A major challenge faced during the preparation of solid polymer nanospheres for advanced drug delivery is the ability to generate reproducible near-monodisperse polymer nanospheres having the desired matrix structure and surface morphology (Bhatt and Shah 2012; Sackmann et al. 2014; Zhang et al. 2012). Solid polymeric nanospheres have received considerable attention due to their potential applications. These include therapeutic agents, such as proteins, genes and drugs (Bourges et al. 2003; Capretto et al. 2012; de Jalón et al. 2001; Hall et al. 2007; Mundargi et al. 2008), disease detection and therapy (Byrne et al. 2008), multimodal contrast enhancement (Kim et al. 2010; Pisani et al. 2008; Schneider et al. 1992; Xu et al. 2009), cell/enzyme experiments, targeted therapeutic applications (Fernandez-Fernandez et al. 2011; Gao et al. 2008; Xu et al. 2011), chemical reagents (Meier 2000; Yu et al. 2011) and controlled delivery (Zhang et al. 2012). In order to conceive polymer nanospheres with a desired structure, numerous techniques including emulsion polymerization, suspension polymerization (Jahn et al. 2008; Liu et al. 2010; Shestopalov et al. 2004; Song et al. 2006), spray drying (Vehring 2008), phase separation (Chan et al. 2005; Chang et al. 2010), electrohydrodynamic techniques (Eltayeb et al. 2013b; Jayasinghe et al. 2004; Nangrejo et al. 2008), self-assembly (Chan et al. 2005; Cui et al. 2011; Shestopalov et al. 2004) as well as microfluidics (Sun et al. 2013) have been developed over the past few decades.

A popular method is microfluidics widely used in the preparation of polymer nanospheres due to the fact that microfluidic technologies offer compelling advantages, including cost-effective preparation and easy and effective control of fluid flow over the other methods (Seiffert 2011; Stride et al. 2008). Several microfluidic methods with different device geometries, including T-junctions, flow focusing devices and co-flow or cross-flow capillaries for generating continuous droplets and subsequently polymer nanospheres, have been described in the literature (Dendukuri and Doyle 2009; Köhler et al. 2011; Liu and Qin 2013; Song et al. 2010; Wang 2013; Xu et al. 2012). In particular, droplet-based microfluidic methods have been widely used to prepare discrete and independently controllable droplets leading to polymer nanospheres with various geometries and polydispersity (Kamio et al. 2008; Serra and Chang 2008; Song et al. 2010).

Polymethylsilsesquioxane (PMSQ) has been used as a model micro/nanosphere material due to its interesting chemical, physical, drug release and biocompatibility properties (Quintanar-Guerrero et al. 1998; Xu et al. 2005). Studies conducted by Ye et al. (2010) using a microfluidic technique have shown that solid PMSQ microspheres 28 µm in diameter have been produced via monodisperse droplet generation. In addition, Chang et al. (2010) used the process of co-axial electrohydrodynamic atomization to prepare submicrometer capsules using PMSQ and a volatile liquid, perfluorohexane (PFH).

Solvent volatility has an influence on the preparation of polymer nanospheres with an enhanced surface roughness (Arshady 1991). In order to enhance the desired matrix structure and surface morphology, a volatile liquid, PFH has been used as an excipient in microfluidic techniques due to its very limited solubility and miscibility with organic solvents and most compounds, and very low toxicity which is preferred in the encapsulation of hydrophilic and lipophilic drugs (Kucuk et al. 2014; Mana et al. 2007). Kucuk et al. (2014) reported that having a tailored rough surface on the polymer nanospheres resulted in increased drug accessibility to the release medium and thus correlated with a higher initial burst release. It is clear that the aforementioned properties and applications confirm that PFH is a suitable excipient in terms of drug delivery requirements to generate polymeric nanospheres.

In this work backed by high speed camera footage, we used a V-shaped microfluidic junction device to generate near-monodisperse polymer nanospheres from droplets and investigated how system parameters (flow rates of PMSQ and PFH) and solution properties influenced the sphere size and surface roughness in a one-step process.

## Materials and methods

### Materials

PMSQ powder, average molecular weight 7,465 g mol^−1^, was purchased from Wacker Chemie AG, GmbH, Burghausen, Germany. Liquid PFH was provided by F2 Chemicals Ltd., Lea, UK (purity grade, 99.7–100 %; density, 1,710 kg m^−1^). Ethanol was procured from the Sigma-Aldrich (Poole, UK; purity grade, 99.7–100 %; density, 790 kg m^−1^).

### Solution preparation

5, 10, 20, 30, 40 and 50 wt% PMSQ was dissolved in ethanol in a sealed vial for 1,800 s at ambient temperature (23 ± 2 °C), using a magnetic stirrer.

### Characterisation of solutions

The standard data sheet of F2 Chemicals Ltd. provided the physical properties of PFH. The polymer solutions were characterised to measure surface tension, viscosity and density using calibrated equipment. A VISCOEASY-L rotational viscometer (Schott GERÄTE GMBH, Germany) and an Ostwald U-tube viscometer were used to measure the viscosity. A tensiometer K9 (Kruss GmbH, Germany, standard Wilhelmy plate method) was used to determine the surface tension. A standard 25-ml density bottle was used to measure the density. All experiments were conducted at the ambient temperature (23 ± 2 °C), and ethanol was utilized as a cleaning and standardising agent prior to characterisation experiments.

### Preparation of nanospheres

**Fig. 1:**
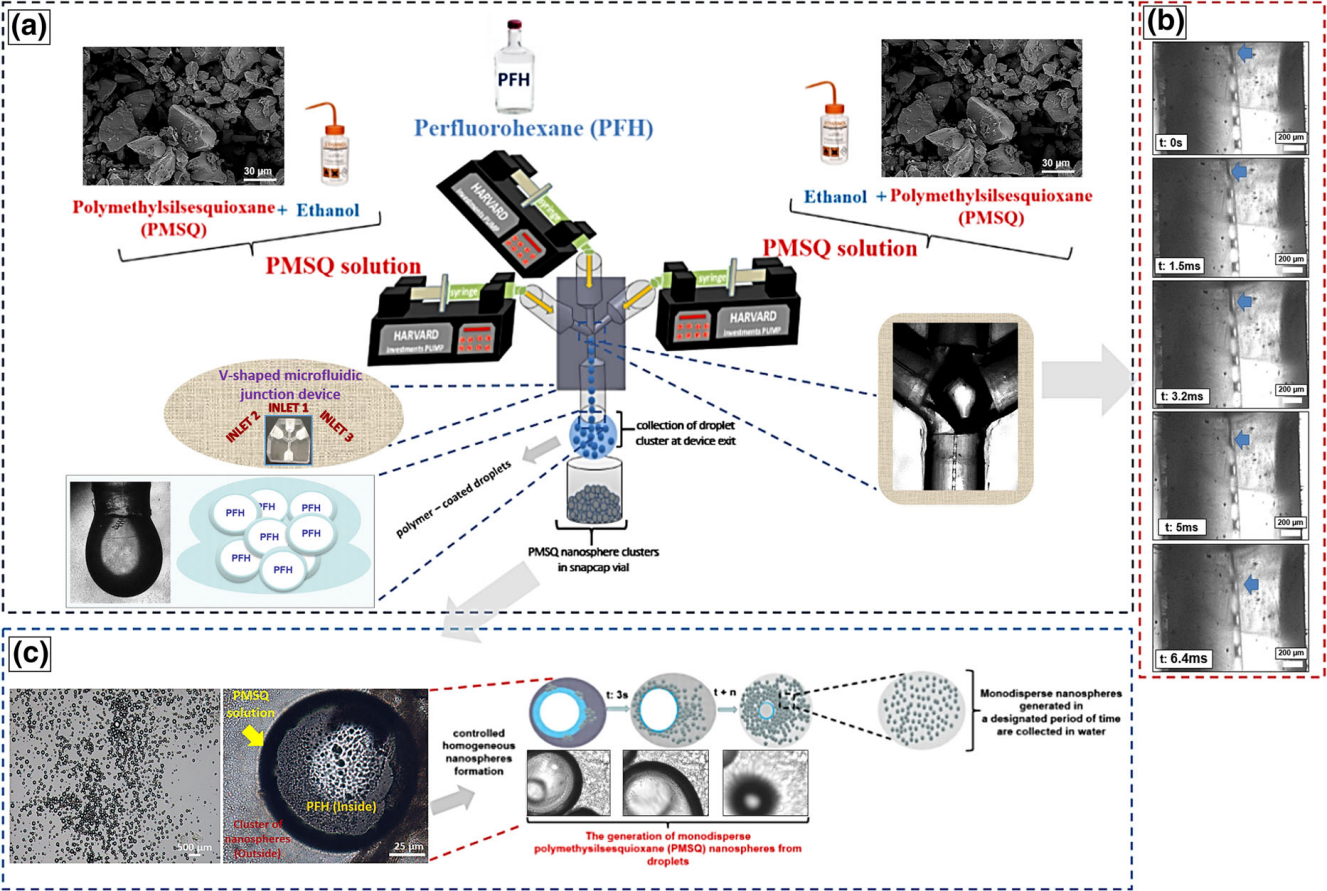
Solid polymer nanosphere preparation using V-shaped channels of the microfluidic junction: **a** apparatus schematic, **b** high speed camera frames of PFH filled PMSQ droplet generation and **c** optical images of the PMSQ polymer nanosphere formation. *t* denotes times

A transparent V-shaped microfluidic junction (VMJ) device was designed and constructed using polymethylmethacrylate (PMMA) with dimensions of 22 × 27 × 15 mm and was used to prepare the polymer nanospheres. Teflon-fluorinated ethylene polypropylene (TEP) capillaries with internal and external diameters of 100 µm and 1.6 mm, respectively, were used to provide continuous flow of the PMSQ solutions (5–50 wt%) and PFH from high precision pumps (Harvard PHD 4400, Apparatus, Edenbridge, UK) to the VMJ device. A schematic illustration of the preparation of solid polymer nanospheres is depicted in Fig. 1. As shown, the PMSQ solutions and PFH are fed from 10-ml plastic syringes (Becton Dickinson, Oxford, UK) using the high precision pumps and the V-shaped channels of the microfluidic junction (Fig. 1a). All liquids mixed in the centre of the device where the channels of the microfluidic junction meet. Subsequently, formation of droplets occurred. These resultant droplets are then guided down an exit channel (outlet capillary) placed at the bottom, and droplet clusters are collected at the channel exit (Fig. 1b). Upon impact with the water in the collector, the droplet is disrupted and releases the volatile solvent while the polymeric material forms nanospheres (Fig. 1c). Resulting nanospheres were collected in a glass vial filled with distilled water.

Optimization studies were conducted to obtain monodisperse nanospheres by varying the polymer (PMSQ) concentration (5–50 wt%), the flow rate of the PMSQ solutions and of the PFH (in the range 50–300 µl min^−1^). The flow processes were observed using a Phantom V7 high speed camera (provided by Engineering and Physical Science Research Council of the UK).

### Characterisation of nanospheres

Droplets were observed using a Nikon Eclipse ME-600 optical microscope (Nikon Co, Tokyo, Japan) as soon as they were generated. Samples of collected spheres were left to dry for 48 h at the ambient temperature (23 ± 2 °C) in a desiccator. They were then sputter coated for 200 s to apply a thin layer of gold to prepare them for SEM imaging (5 kV). A JEOL JSM 6301 F SEM was used to characterise the size and morphology of the produced nanospheres. 200 nanospheres were studied using image analysis software (ImageJ 1.47n, Wayne Rasband National Institute of Health, USA).

Transmission electron microscopy (TEM, JEOL JEM 1010) was used to characterise the internal structure of the nanospheres. For TEM, the collected nanospheres were suspended in distilled water and placed on a copper grid (provided by Agar Scientific Ltd., Stansted, UK).

Atomic force microscopy (AFM) was used to investigate the surface of the produced nanospheres. The images were obtained by scanning the resulting spheres kept on a mica surface in air under ambient conditions using an AFM (Bruker Multimode 8.0, Santa Barbara, CA, USA; Veeco Nanoscope analysis software Version V 6.14r1) operated using the tapping mode. Dried samples were scanned by Bruker silicon nitride tips with a force constant of 0.12 N m^−1^ at 1 Hz with a resolution of 512 × 512 pixels for all images. To avoid structural changes of the sample, the tip loading force was minimized.

## Results and discussion

### Mechanism of nanosphere formation

In this study, PMSQ polymer nanospheres have been obtained using a V-shaped microfluidic junction (VMJ) device when a steady continuous stream of droplets was first attained (see supplementary information S1 provided). High speed camera images of the sequence of droplet formation in the VMJ device show that a droplet is generated every 6.4 ms (Fig. 1b). In addition, Fig. 1b shows necking of a spherical droplet to break off at the top of the outlet capillary when both PMSQ and PFH flowed in at 300 µl min^−1^. The two immiscible liquids are infused into the mixing area in order to generate droplets and the less dense liquid encapsulates the other. This could be due to the fact that the PMSQ solution surface tension for all concentrations was higher than PFH (Table 1). Thus, PMSQ is infused into the mixing area and acts as the driving force responsible for encapsulating the PFH.

**Table 1:** Physical properties of PFH and various PMSQ solutions used in this work (mean ± standard deviation, *n* = 5)

Materials	Density (kg m^−3^)	Viscosity (mPa s)	Surface tension (mN m^−1^)
PFH	1,710 (±5.1)	1.1 (±0.11)	12 (±1.1)
PMSQ 5 wt%	762 (±5.0)	1.0 (±0.10)	21 (±1.0)
PMSQ 10 wt%	791 (±5.2)	1.3 (±0.09)	21 (±1.2)
PMSQ 20 wt%	807 (±4.7)	1.9 (±0.12)	23 (±1.0)
PMSQ 30 wt%	836 (±5.3)	3.1 (±0.11)	23 (±1.1)
PMSQ 40 wt%	871 (±5.1)	5.4 (±0.10)	23 (±1.2)
PMSQ 50 wt%	952 (±4.9)	5.6 (±0.09)	25 (±0.9)

The generated encapsulated droplets stream down through the outlet capillary and were gathered in insoluble media at the channel exit (see supplementary information S2 provided). Upon making contact with an aqueous environment, (distilled water in collecting vial) sphere generation from these droplets becomes evident. Under an optical microscope at a post-collection time of approximately 100 s, the resultant droplets were approximately 120 µm in diameter (Fig. 1c). A cluster of spheres is seen on the droplet surface (Fig. 1c). Upon impact with the water in the collector, the droplet breaks up much like an explosion to release the PFH solvent, while the PMSQ coating forms nanospheres. The high density of spheres on the surface is brought about by the spontaneous bursting of the droplet surface. Evaporation of the PFH continues and the nanospheres shrink and adopt a rough surface (see supplementary information S3 provided). This stage leads to solidification. Eventually, PMSQ polymer nanospheres with diameter in the range of 80–920 nm were obtained.

### Influence of polymer concentration

**Fig. 2:**
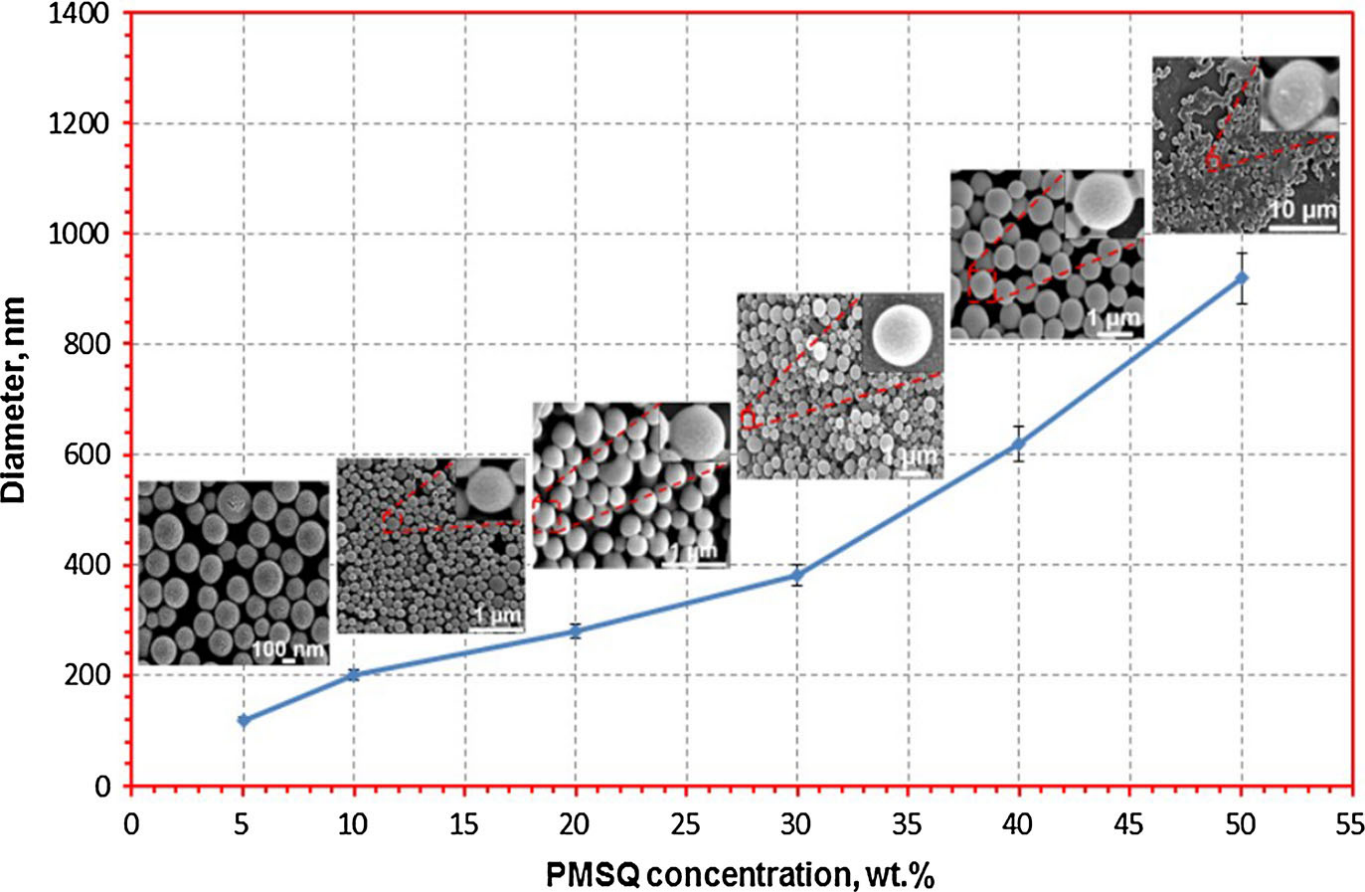
A graph showing diameter of nanospheres as a function of PMSQ concentration (5–50 wt%) at a constant PFH and PMSQ flow rate (300 µl min^−1^). The *insets* are SEM images of PMSQ nanosphere surfaces corresponding to each polymer concentration. *Error bars* show standard deviation of the diameters

The size and surface morphology of polymer nanospheres were influenced by the concentration of the polymer solution. As shown, when the concentration of PMSQ solution was decreased in the range of 50–5 wt%, the diameter of PMSQ nanospheres decreased significantly from 920 to 80 nm, respectively (Fig. 2). Moreover, this decrease of polymer concentration affected the polydispersity of the PMSQ nanospheres, decreasing from 19 to 11 %, respectively. As can be seen from the SEM images (inset in Fig. 2), PMSQ nanospheres were spherical but their surface roughness changed with polymer concentration, and at the lowest (5 wt%) a rough surface was clearly seen (Fig. 2). This could be due to the fact that a change in the concentration of polymer has an effect on its physical properties such as surface tension, viscosity and density, as shown in Table 1. An increase in the polymer concentration leads to a clear increase in viscosity, with more subtle changes to the surface tension. Two major physical properties of solutions which affect sphere generation are surface tension and viscosity, both of which can be influenced by polymer concentration (Enayati et al. 2010; Ghanbar et al. 2013; Kucuk et al. 2014). When there is a decrease in the surface tension of the solution, in general, a decrease in the average sphere size can also be observed (Craig et al. 1993; Eltayeb et al. 2013a). Moreover, the viscosity of the solutions varies considerably for all the PMSQ solutions and a viscosity <100 mPa s is necessary for droplet formation (Liu and Hsieh 2002). An increase in the concentration of PMSQ results in an increase in the density and viscosity, and thus, is expected to increase the sphere size (Kucuk et al. 2014). Studies conducted by Kolishetti et al. (2010) also reported that the polydispersity indices of poly(d,l-lactic acid-*co*-glycolic acid)-block-poly(ethyleneglycol) copolymer (PLGA-PEG) nanospheres produced by the hydrodynamic flow focusing method was in the range 6–17 %. The findings of the current study are comparable with previous research focusing on the preparation of nanospheres using PMSQ (Chang et al. 2010). Although they used a different method, Chang et al. (2010) found that an increase in PMSQ solution concentration (18–36 wt%) led to an increase in the hollow sphere size (range 400–600 nm) with a polydispersity index range of 22–30 %. Thus, the literature indicates that an increase in concentration brings about an increase in sphere size regardless of the flow rates in several techniques used to generate spheres. However, concentration can only be increased to a certain extent, where it does not hinder production feasibility due to very high viscosity.

### Effect of flow rate

**Fig. 3:**
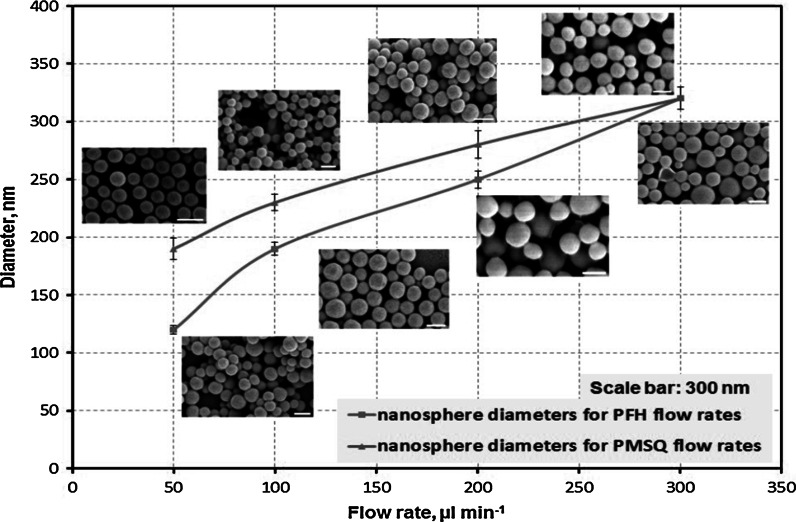
A graph showing diameters of nanospheres for PMSQ flow rates (50–300 µl min^−1^) at a constant PFH flow rate (300 µl min^−1^) (*red line*), and for PFH flow rates (50–300 µl min^−1^) at a constant PMSQ flow rate (300 µl min^−1^) (*blue line*). *Error bars* show standard deviation of the diameters. The *insets* are SEM images of PMSQ nanosphere surfaces corresponding to flow rates of PMSQ and PFH. (Color figure online)

Flow rates of PMSQ and PFH had a significant influence on the sphere size (Fig. 3). Varying the flow rates from 50 to 300 µl min^−1^ for either PFH or PMSQ while keeping the other constant resulted in increased nanosphere diameter in both cases. An increase in diameter from a minimum of 120 nm to a maximum of 320 nm for PFH is detected from the graph, while a similar gradual increase from a minimum of 190 nm to a maximum of 320 nm is also observed when PMSQ flow rates were varied within the same flow rate range (Fig. 3). Findings are further confirmed by the SEM images, inserted in Fig. 3. The SEM images present near-monodisperse nanospheres with spherical morphology as a result of the changes in the flow rates. The change in size could be as a result of increase in the PMSQ solution flow rate which induces stronger shear forces and/or increase the volume fraction of material flow per unit time, such that larger nanospheres are formed. The findings of the current study are comparable with the previous research on the preparation of microspheres using PMSQ and PFH (Chang et al. 2010; Kucuk et al. 2014). To identify the influence of the polymer solution flow rate, Rondeau and Cooper-White (2008) prepared alginate spheres via a microfluidic technique with sizes ranging from 10 to 300 nm under the influence of flow rates in the range of 0.08–0.8 µl min^−1^ Recently, studies conducted by Valente et al. (2012) using a confined impinging jet mixer have shown that an increase in PEGylated solution flow rate (5–120 µl min^−1^) led to an increase in the sphere size from 160 to 350 nm. Although they used a different technique, Chang et al. (2010) reported that coaxial electrohydrodynamic atomization (CEHDA) allows the production of capsules using PMSQ in the range of 275–660 nm in diameter at the PMSQ flow rates of 200–600 µl min^−1^. However, the CEHDA technique is not capable of encapsulating the PFH liquid in the PMSQ solution always because a ‘stable jet’ could not be achieved for PMSQ flow rates <200 µl min^−1^ (Chang et al. 2010). In sharp contrast, studies performed by Ghanbar et al. (2013) reported large diameters ranging from 150 to 300 µm under the effect of PLGA solution flow rates of 30–200 µl min^−1^ using a one-step electrohydrodynamic atomization and thermally induced phase separation (TIPS) method used to produce PLGA porous microspheres. These findings show that there is an impact of polymer solution flow rate on the ultimate sphere size; the influence is quite strong at low flow rate; however, the impact for values greater than 20–40 ml min^−1^ becomes less pronounced, particularly for nanospheres.

**Fig. 4:**
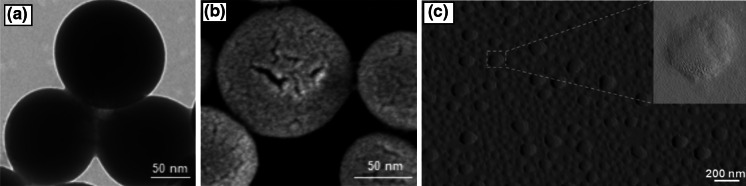
**a** Transmission electron, **b** scanning electron and **c** atomic force micrographs of the solid polymer nanospheres showing their interior and surface morphology. PFH flow rate of 50 µl min^−1^ and a PMSQ flow rate of 300 µl min^−1^ were used at the lowest PMSQ concentration (5 wt%)

Figure 3 also describes the relationship between the flow rate of the PFH and the mean diameter of the nanospheres. The flow rate at inlet (PFH) was varied from 50 to 300 µl min^−1^, while keeping the flow rate of PMSQ solution via inlet 2 and 3 at 300 µl min^−1^. It is clear that an increase in the PFH flow rate results in an increase in the mean diameter of nanospheres. The SEM images confirm that the PMSQ nanospheres generated were nearly spherical in shape in spite of the changes in the flow rates (Fig. 3). Electron microscopy studies of the nanospheres are shown in Fig. 4. Transmission electron microscope images showed that the interior structure of the nanospheres is solid (Fig. 4a). In addition, scanning electron microscope images depicted the surface of the nanospheres contains fine pores and cracks (Fig. 4b). Further investigation with atomic force microscopy (AFM) showed the rough surface characteristics of the nanospheres as depicted in Fig. 4c. In addition, Fig. 4c inset shows the surface morphology of a nanosphere at high magnification. This image clearly shows the numerous undulations on the surface of the nanospheres. This could probably be as a result of the high volatility of PFH which subsequently evaporates from the core of droplets to furnish the surface of the nanospheres. The enhanced surface roughness which prevails has been extremely useful to anchor drugs such as itraconazole and this work is described in a separate paper (Kucuk et al. 2014). Our findings also indicate that the effect of PFH flow rate on the final sphere size and surface morphology is more prominent.

## Conclusions

Solid polymer nanospheres have been conceived using a V-shaped microfluidic junction device. The device used in this work offers a simple method to prepare nanospheres from polymeric droplets. It also enables optimization of nanosphere size. The sphere diameters obtained ranged from 80 to 920 nm, (polydispersity index: 11–19 %) and at the lowest PFH flow rate of 50 µl min^−1^, nanospheres of 120 nm diameter were generated. The solution properties (polymer concentration) and the process parameters, such as PMSQ solution and PFH flow rates, have a significant effect on the sphere diameter and characteristics, such as surface roughness, which is desirable for some therapeutic applications such as drug delivery. In current work, we are using other biodegradable polymer systems to make this processing and forming method even more generic and versatile. We are also working towards optimizing the process parameters in order to further control the polydispersity of the nanospheres and to prepare different types of nanospheres having internal porosity.

## Electronic supplementary material

Below is the link to the electronic supplementary material.

Supplementary material 1 (PDF 80 kb)

Supplementary material 2 (MPEG 5454 kb)

Supplementary material 3 (MPEG 18714 kb)

Supplementary material 4 (MPEG 42198 kb)
