# Supplementary material for: Microfluidic preparation of polymer nanospheres
Source: J Nanopart Res. 2014 Dec 4;16(12):2626. doi: 10.1007/s11051-014-2626-5 (PMC4255063; doi:10.1007/s11051-014-2626-5)
Supplement: Supplementary file 1 — Supplementary material 1 (PDF 80 kb) [file 11051_2014_2626_MOESM1_ESM.pdf]

List of Supplementary Materials

S_1: High speed camera video: Droplet creation and generation in the V-shaped microfluidic junction device.

S_2: High speed camera video: Collection of generated droplets in insoluable media at the outlet.

S_3: High speed camera video: Droplet to nanospheres transformation.
